# Activation of p53 by Chemotherapeutic Agents Enhances Reovirus Oncolysis

**DOI:** 10.1371/journal.pone.0054006

**Published:** 2013-01-16

**Authors:** Da Pan, Paola Marcato, Dae-Gyun Ahn, Shashi Gujar, Lu-Zhe Pan, Maya Shmulevitz, Patrick W. K. Lee

**Affiliations:** 1 Department of Microbiology and Immunology, Dalhousie University, Halifax, Canada; 2 Department of Pathology, Dalhousie University, Halifax, Canada; 3 Department of Medical Microbiology and Immunology, University of Alberta, Edmonton, Canada; Virginia Commonwealth University, United States of America

## Abstract

Mammalian reovirus is a benign virus that possesses the natural ability to preferentially infect and kill cancer cells (reovirus oncolysis). Reovirus exploits aberrant Ras signalling in many human cancers to promote its own replication and spread. *In vitro* and *in vivo* studies using reovirus either singly or in combination with anti-cancer drugs have shown very encouraging results. Presently, a number of reovirus combination therapies are undergoing clinical trials for a variety of cancers. Previously we showed that accumulation of the tumor suppressor protein p53 by Nutlin-3a (a specific p53 stabilizer) enhanced reovirus-induced apoptosis, and resulted in significantly higher levels of reovirus dissemination. In this study, we examined the role of p53 in combination therapies involving reovirus and chemotherapeutic drugs. We showed that sub-lethal concentrations of traditional chemotherapy drugs actinomycin D or etoposide, but not doxorubicin, enhanced reovirus-induced apoptosis in a p53-dependent manner. Furthermore, NF-κB activation and expression of p53-target genes (*p21* and *bax*) were important for the p53-dependent enhancement of cell death. Our results show that p53 status affects the efficacy of combination therapy involving reovirus. Choosing the right combination partner for reovirus and a low dosage of the drug may help to both enhance reovirus-induced cancer elimination and reduce drug toxicity.

## Introduction

Mammalian reovirus is a benign virus that preferentially replicates in cancer cells while sparing normal cells (reovirus oncolysis) [1], [2]. Cancer cells harboring aberrant Ras-signaling pathways have been shown to promote reovirus (Strain T3D) infection and virus spread by affecting various steps of the reovirus replication cycle [3]–[6]. Considering the prevalence of aberrant Ras signalling in human cancers, reovirus has been explored as a potential cancer therapy. Numerous studies have indicated that reovirus can induce regression of different types of cancer *in vitro* and *in vivo* [7]–[21]. Reovirus (Reolysin™) is now undergoing phase II/III clinical trials as a single therapy or combination therapy with various chemotherapy drugs. These studies showed low toxicity profile of reovirus and patients’ partial to complete response to reovirus [8], [10], [11], [20], [21], suggesting that reovirus is a promising alternative cancer therapy.

The majority of traditional chemotherapy agents target fast-growing tumor cells based on the rationale that cancer cells are rapidly dividing and are therefore more sensitive to drugs that affect DNA replication. Since ideally, most normal cells are quiescent and rest in the G_0_/G_1_ phase of the cell cycle, chemotherapy drugs that damage dividing cells in the S or M phase would preferentially target tumor cells. Drugs that induce DNA damage (resulting in single or double strand breaks) will initially activate the cellular DNA repair response, but prolonged exposure to these chemotherapy drugs will induce apoptosis in fast dividing tumor cells, resulting in tumor mass reduction. Unfortunately, these chemotherapy drugs also damage fast growing normal cells, causing major side effects to patients. Indeed, emerging evidence shows that traditional chemotherapy drugs have reached their plateau of efficacy as the primary modes of treatment, whereas combination therapies coupling these drugs with specific inhibitors or alternative therapies are rapidly gaining recognition in cancer treatment [22].

The tumor suppressor protein p53 plays a vital role in dictating the fate of a cell upon exposure to stress or genomic damage. Many commonly used chemotherapy drugs such as pyrimidine analogs and topoisomerase II inhibitors induce cellular DNA damage response and inevitably activate wild-type p53. Our group previously showed that a specific p53 stabilizer (Nutlin-3a) significantly enhanced reovirus-induced apoptosis [23]. In the present study, we combined reovirus with three currently used anti-cancer drugs (actinomycin D, etoposide, and doxorubicin) that are known to activate p53, and examined their effects on cancer cell killing. We observed that sub-lethal concentrations of actinomycin D or etoposide, but not doxorubicin, enhanced reovirus-induced apoptosis in a p53-dependent manner. The enhancement of apoptosis was accompanied by increased NF-κB activation and was dependent on the expression of p53 target genes such as *p21* and *bax*. Our results show that the p53 status in a cancer can affect the outcome of combination therapy with reovirus and chemotherapy drugs. Choosing the right drug partner for reovirus can both enhance reovirus-induced cancer cell death but also potentially reduce the side-effects of the drug.

## Materials and Methods

### Cell Lines Reovirus and Reagents

HCT116 cells (p53+/+, p53−/−, p21−/−, PUMA−/−, Bax−/−, PUMA−/−p21−/− and Bax−/−p21−/−) were from Dr. Bert Vogelstein (John Hopskins University, Baltimore, MD) [24]–[26]; L929 and human colon adenocarcinoma DLD1 cells were purchased from and cultured as instructed by American Type Culture Collection (Manassas, VA).

Lentivirus vector pLKO.1-puro (control) and Noxa-kd plasmids were purchased from Sigma-Aldrich (TRCN0000150554 for Noxa-kd1 and TRCN0000150555 for Noxa-kd2). Lentivirus clones (including control, Noxa-kd1 and Noxa-kd2) were produced to infect HCT116 p53+/+ cells. Stable cells lines were created by selection with puromycin. Retroviruses containing either scrambled sequence or p53 specific shRNAmir sequence (control and p53kd) in pSMN construct from Open Biosystems (Thermo Fisher Scientific Inc., Huntsville, AL) were prepared as described before (23).

Reovirus (Serotype 3 Dearing, T3D) was propagated in a L-929 suspension culture in JMEM (Sigma-Aldrich Canada, Oakville, ON, Canada) supplemented with 5% horse serum, 1% Anti-Anti (AA) and sodium pyruvate (110 mg/L). Reovirus was purified following established procedures [27]–[29]. Reovirus activity was determined by standard virus plaque titration.

Nutlin-3a was provided by Hoffmann-La Roche Inc. (Nutley, NJ, USA). Actinomycin D (ActD), etoposide (Etp) and doxorubicin (Dox) were purchased from Sigma-Aldrich Canada. Caspase inhibitor I Z-VAD(OMe)-FMK (ZVAD) and InSolution™ NF-κB activation inhibitor (Inhibitor N) were purchased from Calbiochem (Merck in Canada, Toronto, ON, Canada).

Antibodies against p53 (Do-I), β-actin and NF-κB p65 sub-unit were purchased from Santa Cruz Biotechnology, Inc. (Santa Cruz, CA).

### Immunostaining for NF-κB p65 Nuclear Translocation

HCT116 cells grown overnight on gelatin-coated coverslides were treated with appropriate concentration of a specific chemotherapy drug, reovirus or the combination of reovirus and the specific drug. At 12 hour-post-infection (hpi), cells were fixed and then stained with anti-p65 (Santa Crutz), anti-reovirus antibodies followed by Alexa Fluor® 488 F(ab')_2_ fragment of goat anti-rabbit IgG (H+L), Cy3 AffiniPure Goat Anti-Mouse IgG (H+L) (Jackson ImmuResearch Laboratories, Inc., West Grove, PA) and To-Pro-3 (Invitrogen) for nuclear staining. Images were captured with a Zeiss LSM 510 laser scanning confocal microscope.

### Apoptosis Assay, Sub-G1 Profiling and Fluorescence Activated Cell Sorting (FACS) Analysis

At 24 hpi, cells were harvested and stained with an apoptosis marker Annexin V-FITC and a viability marker 7-AAD (BD pharmingen, Oakville, ON, Canada) according to manufacture’s instructions [30], [31]. Cell death was quantified by flow cytometry using a FACScan flow cytometer (BD Biosciences, San Jose, CA). To determine the percentage of infected cells, cells were fixed by 4% paraformaldehyde on ice at indicated time. Cells were then washed with PBS and stained with anti-reovirus antibody followed by properly conjugated secondary antibody. One million cells were sorted, quantified by FACS and analyzed using WinMDI Version 2.8.

For sub-G1 profiling, cells fixed with 70% ice-cold ethanol were washed with PBS and then stained with Propidium Iodide (PI, Sigma-Aldrich, St. Louis, MO) staining buffer (50 µg/mL of PI, 20 µg/mL RNase A and 0.5% BSA) for 30 minutes. Cells were washed twice with cold PBS+10% Fetal bovine serum (FBS) and two million cells were sorted and quantified by FACS and analyzed using WinMDI Version 2.8.

### RNA Extraction and Real-time Quantitative Polymerase Chain Reaction (real-time qPCR)

RNAs extracted with TRIzol® and purified with PureLink™ RNA mini column (Invitrogen) were reverse transcribed using the Superscript II reverse transcriptase kit (Invitrogen). QuantiFast SYBR RT-PCR kit (Qiagen, Mississauga, ON, Canada) with gene-specific primers as described before [23] was used to quantify gene expression as per manufacture’s instruction. Standard curves were generated to calculate relative level of mRNA and GAPDH was used as an internal control.

### Cell Viability Assay

Promega CellTiter 96^®^ AQ_ueous_ Non-Radioactive Cell Proliferation Assay (Promega) was used to determine the viability of chemotherapy drug-treated cells according to manufacture’s instructions. Specifically, 1.5*10^4^ cells/well of HCT116 (p53+/+ or p53−/−) or DLD1 cells were seeded onto 96-well plates at a volume of 100 µL. Serial dilutions of a specific chemotherapy drug were used to treat HCT116 cells at 24 hours post seeding. MTS (3-(4,5-dimethylthiazol-2-yl)-5-(3-carboxymethoxyphenyl)-2-(4-sulfophenyl)-2H-tetrazolium, inner salt) and PMS (phenazine methosulfate) were appropriately mixed and added to the cell culture at 48 h post treatment. DLD1 cells (control or p53kd) were infected with reovirus at MOI of 100 with or without being treated with a specific concentration of a chemotherapy drug. MTS and PMS were added to the cells at 36 hpi. The plates were then incubated at 37°C in a humidified, 5% CO_2_ atmosphere for 1 hour (h) before being read at 490 nm by an ELISA microplate reader. Background absorbance was subtracted as instructed by manufacture instruction.

### Plaque Size Assay

Cells cultured to 90% confluency were infected with serial dilutions of reovirus in a volume of 100 µL in 12-well plates for 1 h at a 37°C and 5% CO_2_ atmosphere. FBS and AA were supplemented to 2 x MEM (modified Eagle’s medium) to a final concentration of 10% FBS and 2 × AA to complete the 2 × MEM mixture. After 1 h of reovirus attachment, the 100 µL of reovirus solutions was removed and an equal volume of 2 × MEM mixture and 2% agar were mixed and applied to cells with or without the proper concentration of chemicals. After the mixture solidified, plates were placed with the solid agar side up in a 37°C incubator for 7 days. Cells were then fixed with 10% formaldehyde for 10 min and the agar layer was taken off. Cells were fixed with methanol for 5 min, washed with PBS three times and incubated with blocking buffer (PBS supplemented with 5% BSA and 0.1% Triton X-100) for 1 h at RT. Rabbit polyclonal anti-reovirus antibody was diluted with blocking buffer at a concentration of 1∶10,000 and applied to cells for 1 h at RT after blocking. Cells were washed three times with PBS +0.1% Triton X-100 and secondary antibody with 1∶1000 goat anti-rabbit antibody conjugated with alkaline phosphatase (Jackson ImmunoResearch Laboratories, Ltd., West Grove, PA) was applied to cells. Plates were incubated at RT for 30 min. After three times of washing with PBS +0.1% Triton X-100, reovirus plaques were developed using BCIP/NBT Alkaline Phosphatase Substrate Kit IV (Vector Laboratories Ltd., Burlingame, CA).

## Results

### Low Concentrations of Actinomycin D and Etoposide Induce p53 Accumulation and Enhance Reovirus-induced Apoptosis in a p53-dependent Manner

We previously showed that Nutlin-3a, a specific p53 stabilizer, enhanced reovirus-induced cancer cell death [23]. It was therefore interesting to determine whether the p53 status in cancer cells affects the outcomes of treatments that involve reovirus combined with traditional anti-cancer drugs. Three common traditional chemotherapy agents were initially chosen for such studies: etoposide (Etp), actinomycin D (ActD) and doxorubicin (Dox). Etoposide (also known as VP-16) and doxorubicin (Adriamycin^®^) are topoisomerase II inhibitors that introduce single or double strand breaks in DNA. Actinomycin D forms a stable complex with DNA to block the movement of RNA polymerase [32].

**Figure 1:**
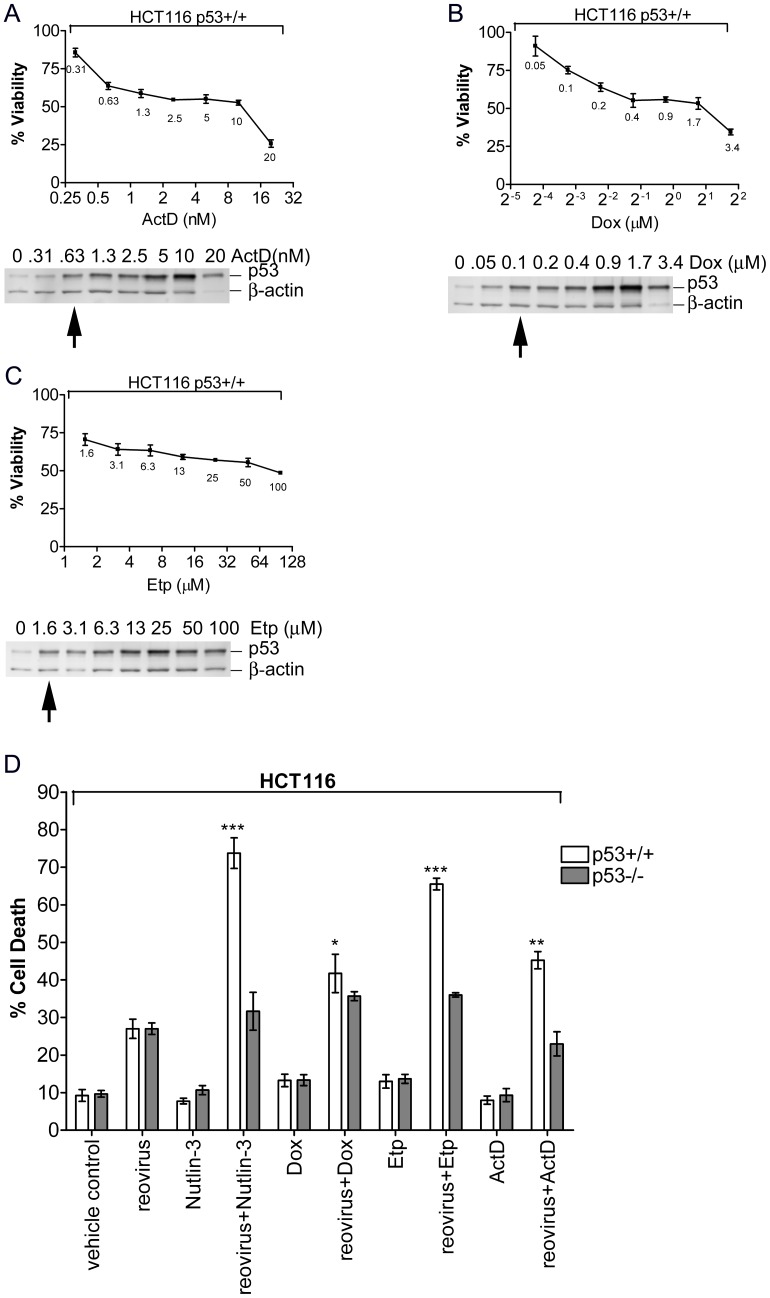
Sub-lethal concentrations of specific chemotherapy drugs enhance reovirus-induced apoptosis. Cell viability of HCT116 p53+/+ cells after exposure to various concentrations of (**A**) ActD, (**B**) Dox or (**C**) Etp. MTS reagents were added at 48 h post treatment. Accumulation of p53 was determined by western blot analysis using p53-specific antibody. Representative data from two independent experiments (triplicate wells, *±s.e.m.*) is shown. **D**. Apoptosis induced by reovirus alone, sub-lethal concentrations of ActD/Etp/Dox alone (Nutlin-3 as control), or the combination of reovirus and the drug. HCT116 p53+/+ and p53−/− cells were infected by reovirus at an MOI of 1 for 1 h and then supplemented with media with or without low concentrations of Dox, Etp or ActD. 1/10,000 dilution of DMSO in cell culture media was used as vehicle control. Percentage of apoptotic cells was determined by double-staining with Annexin V and 7-AAD at 24 hpi. Student’s *t*-test was used to compare percentage of cell death between reovirus infected cells and cells treated by the combination of reovirus and drugs; *p<0.05; **p<0.001; ***p<0.0001.

Since an objective of this study was to reduce drug toxicity, we first determined the lowest dosages of these drugs that, when used singly, generated minimum cell death while inducing enhanced levels of p53. As shown in Fig. 1A–C, the three drugs tested induced accumulation of p53 in a relatively dose-dependent manner. A low concentration of each drug at which p53 was clearly accumulated while at the same time not causing extensive cell death (>50% cell viability) was chosen for further study: Etp (1.6 µM), ActD (0.63 nM), or Dox (0.1 µM).

**Figure 2:**
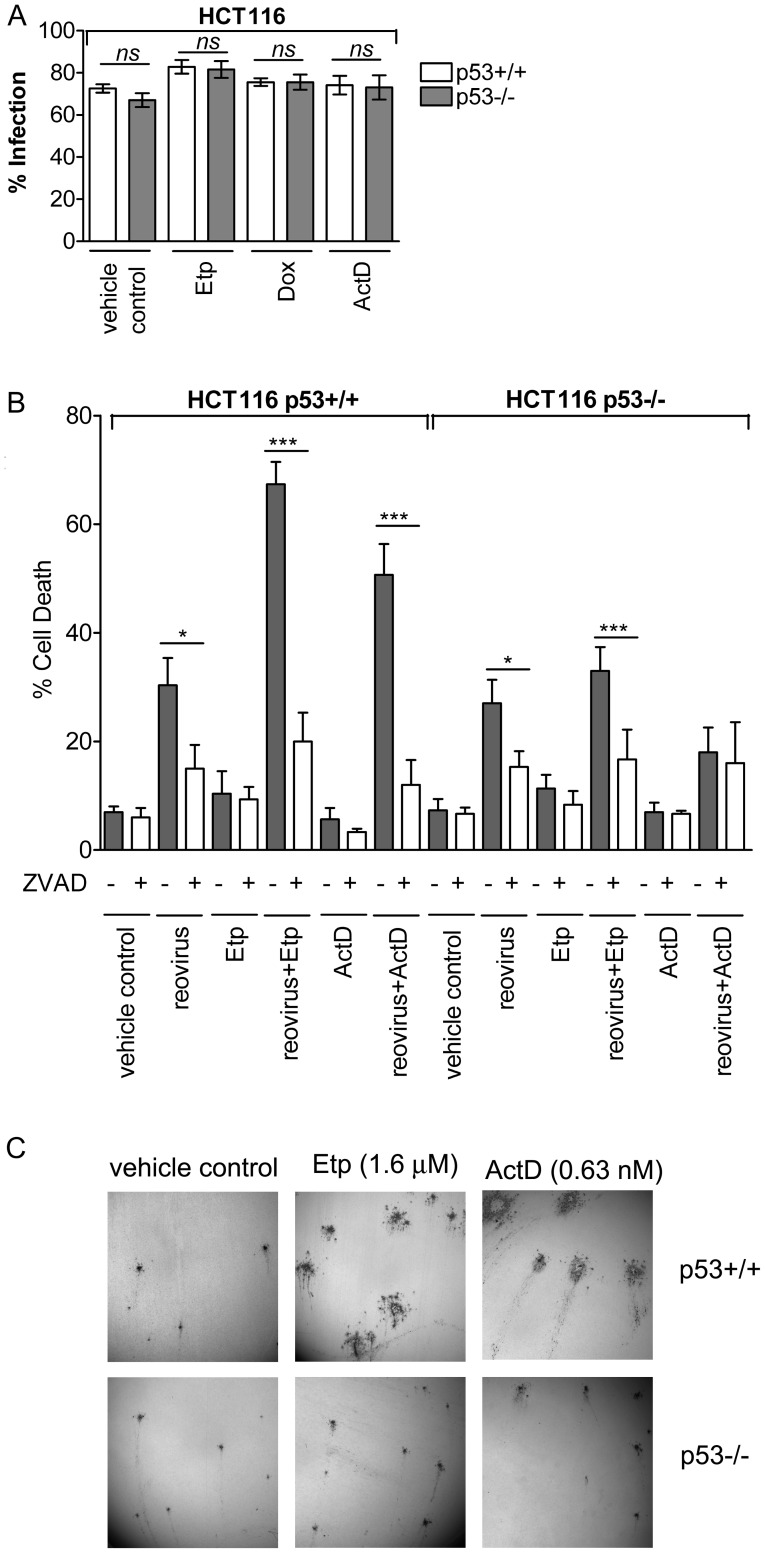
Enhanced apoptosis induced by reovirus and ActD/Etp depends on virus replication and caspase activities. **A.** Percentage of cells that were infected by reovirus with or without the treatment of Dox, Etp, or ActD at 24 hpi, determined by FACS analysis using anti-reovirus antibody. **B.** ***Caspase inhibitor ZVAD blocks the enhancement of cell death induced by the combination of reovirus and a low concentration of ActD or Etp.*** HCT116 p53+/+ and p53−/− cells were treated with ZVAD for 1 h before reovirus infection. Following reovirus infection, ZVAD and a low concentration of ActD or Etp were added to culture media of reovirus-infected cells. Cell death was determined by Annexin V and 7-AAD staining at 24 hpi. Student’s *t*-test was used to compare two groups of data; *p<0.05 and ***p<0.0001. **C**. Immunohistochemical staining of the plaques formed on reovirus-infected HCT116 cells in the presence or absence of ActD (0.63 nM) or Etp (1.6 µM).

We then determined whether the low concentrations of these chemotherapy agents could enhance reovirus-induced cell death and whether the enhancement was p53-dependent by comparing their effects on HCT116 p53+/+ and HCT116 p53−/− cells. As shown in Fig. 1D, none of the three drugs, when used singly at the indicated concentrations, induced significant cell death in p53+/+ or p53−/− cells. Reovirus, in the absence of any drugs, induced comparable levels (∼28%) of apoptosis in p53+/+ and p53−/− cells at 24 hpi. However, when Etp or ActD, but not Dox, was combined with reovirus, the levels of apoptosis were significantly enhanced in HCT116 p53+/+ cells but not in p53−/− cells. This enhancement was not due to increased number of reovirus-infected p53+/+ cells in the presence of the drugs since the percentages of infected cells (p53+/+ or p53−/−) in drug-treated cultures were not significantly higher than those in drug-free cultures (Fig. 2A and Fig. S2). Furthermore, addition of ZVAD to the medium significantly reduced the level of cell death induced by either reovirus alone or the combination of reovirus and ActD or Etp (Fig. 2B), indicating that enhanced cell death induced by the combination treatment is due to caspase-dependent apoptosis. To further determine whether the difference in cell death caused by the combination of reovirus and either ActD/Etp or Dox was cell-type dependent, human colon adenocarcinoma cell line DLD1 cells were treated with retroviruses containing either scrambled sequence or p53 specific shRNAmir sequence (control and p53kd). DLD1 control or p53kd cells were then treated with reovirus with or without the co-treatment of Dox (0.3 µM), Etp (4.8 µM) or ActD (1.89 nM). Slightly higher concentrations of each drug were used for DLD1 than HCT116 cells since they presented higher resistance to drug treatment (data not shown). As shown in Fig. S1A, Etp and ActD, but not Dox, caused significant difference between DLD1 control and p53kd cells in enhancing reovirus-caused cell death. This is consistent with results obtained from HCT116 cells and indicates that certain chemotherapy drugs such as Etp and ActD are more efficient than others (such as Dox) in enhancing reovirus-oncolysis.

Enhanced reovirus-induced cell death in the presence of ActD or Etp was also evident from the significantly larger plaque size compared to untreated cells, reflecting more efficient virus release and cell-to-cell spread (Fig. 2C). ActD or Etp did not enhance the size of plaques produced on p53−/− HCT116 cells, confirming that the enhancement of reovirus dissemination in the presence of these drugs is p53-dependent. (ActD and Etp were chosen for these and subsequent studies due to the clearly manifested p53-dependent apoptosis induced by reovirus when combined with either of these two drugs.).

### Combination of Reovirus with ActD or Etp Enhances Expression of p53-target Genes

**Figure 3:**
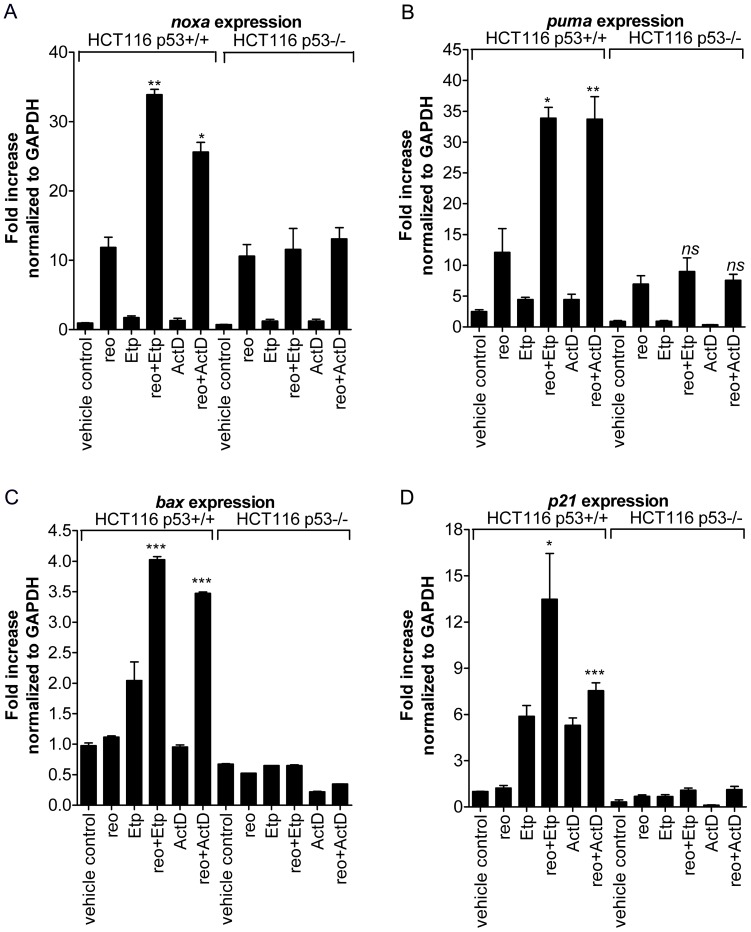
Reovirus combined with ActD/Etp significantly enhances p53-target genes expression. (**A**) *noxa*, (**B**) *puma*, (**C**) *bax* and (**D**) *p21* expression levels in HCT116 p53+/+ and p53−/− cells treated with reovirus (reo), Etp/ActD or the combination of Etp/ActD and reovirus. RNA samples were collected at 24 hpi and processed for real-time q-PCR analysis using gene-specific primers (*±s.e.m.*, n = 3). Student’s *t*-test was used to compare expression levels between reovirus-infected cells and cells treated with the combination of reovirus and a drug; *p<0.05, **p<0.001 and ***p<0.0001.

When a specific p53-stabilizer (Nutlin-3a) was combined with reovirus, the levels of certain p53-target genes such as *puma* and *bax* were further elevated compared to either treatment alone [23]. Therefore, whether levels of these p53-target genes were elevated by the combination of reovirus and ActD/Etp was determined. As shown in Fig. 3A and 3B, reovirus infection alone induced expression of *noxa* and *puma* in a p53-independent manner, consistent with previous observations [23]. However, expression levels of these two genes were significantly enhanced in the p53+/+ cells, but not the p53−/− cells, when reovirus infection was carried out in the presence of ActD or Etp which by themselves had minimal effect on the expression of these two genes. Similarly, ActD and Etp enhanced the expression of *bax* in reovirus-infected p53+/+ cells, but not in p53−/− cells (Fig. 3C). Interestingly, Etp (and to a much lesser extent ActD) also upregulated p21 expression in infected p53+/+ cells (Fig. 3D). Overall, the combination treatment of reovirus and ActD/Etp on p53+/+ cells significantly upregulates p53-target genes compared to drug treatment or reovirus-infection alone.

### Enhancement of Reovirus-induced Apoptosis by ActD/Etp Depends on *p21* or *bax*

**Figure 4:**
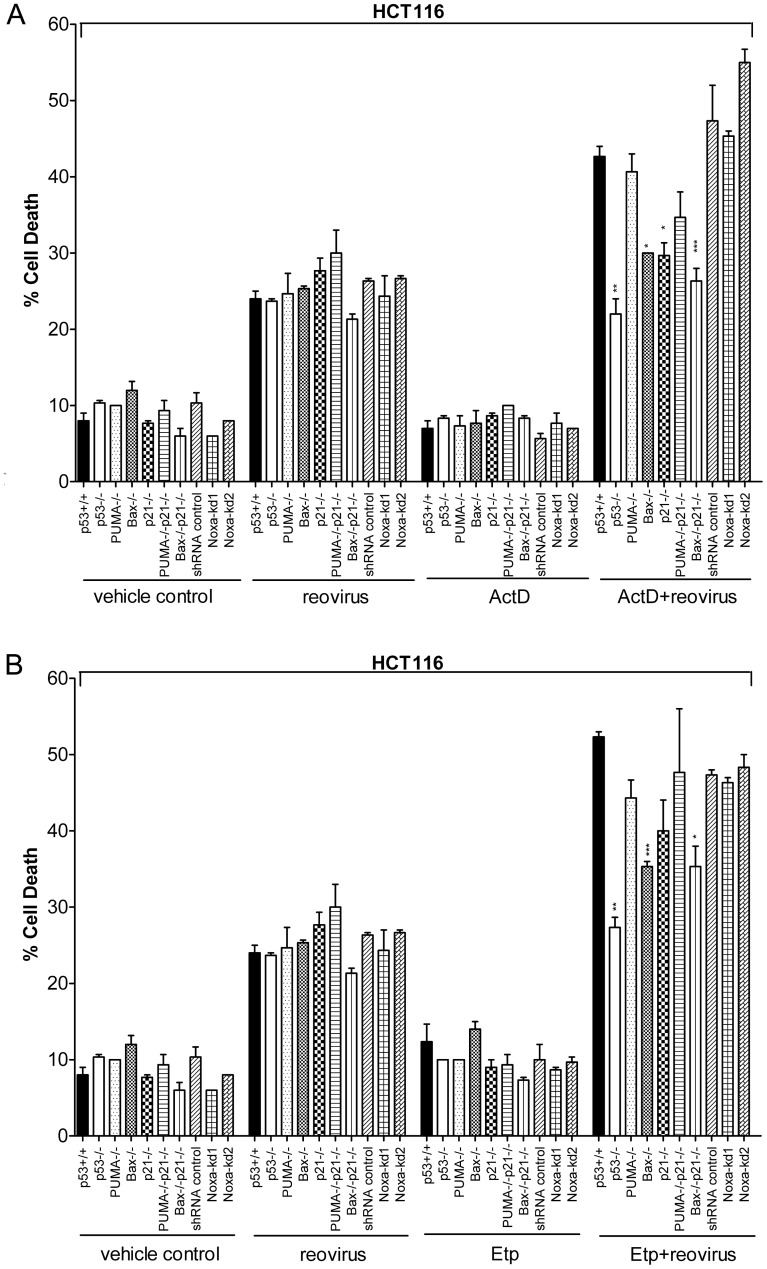
Enhanced apoptosis induced by the combination of reovirus and ActD/Etp requires bax and p21. HCT116 p53+/+ and its isogenic knockout (p53−/−, PUMA−/−, Bax−/−, p21−/−, PUMA−/−p21−/− and Bax−/−p21−/−) cells and HCT116 p53+/+ cells infected with lentivirus containing shRNAs control (shRNA control) or sequences against *noxa* (Noxa-kd1, Noxa-kd2), were infected with reovirus at an MOI of 1 in the presence or absence of (**A**) ActD or (**B**) Etp treatment. Cells were collected at 24 hpi and subjected to Annexin V and 7-AAD staining to determine the percentage of cell death (*±s.e.m.*, n = 3). Student’s *t*-test was used to compare cell death between knockout (or knockdown) cells and p53+/+ cells; *p<0.05, **p<0.001 and ***p<0.0001.

Since various p53-target genes were upregulated by the combination of reovirus and ActD/Etp, it is important to determine which gene(s) are important for the enhancement of apoptosis by the combinations. Therefore, HCT116 p53+/+ cells and its isogenic knockout and knockdown cells (see Materials and Methods and [23]) were infected by reovirus or treated with a combination of reovirus and ActD/Etp. The levels of apoptosis induced by single or combination treatments were determined. As shown in Fig. 4, apoptosis induced by reovirus alone was not significantly affected by loss of *puma*, *bax*, *p21* or *noxa*. However, the enhancement of apoptosis induced by the combination of ActD and reovirus was significantly reduced by the absence of *bax* or when *bax* and *p21* were both knocked out (Fig. 4A). Similar results were obtained with the Etp and reovirus combination (Fig. 4B). In contrast, the status of *puma* or *noxa* did not play a major role in enhancing the apoptosis induced by the combination of reovirus and either of these two chemotherapy drugs. Therefore, *p21* and *bax* seem to be important for the enhancement of apoptosis induced by the combination of reovirus and ActD/Etp.

### Combination of Reovirus and ActD/Etp Induces NF-κB Activation that is Required for Enhanced Apoptosis

**Figure 5:**
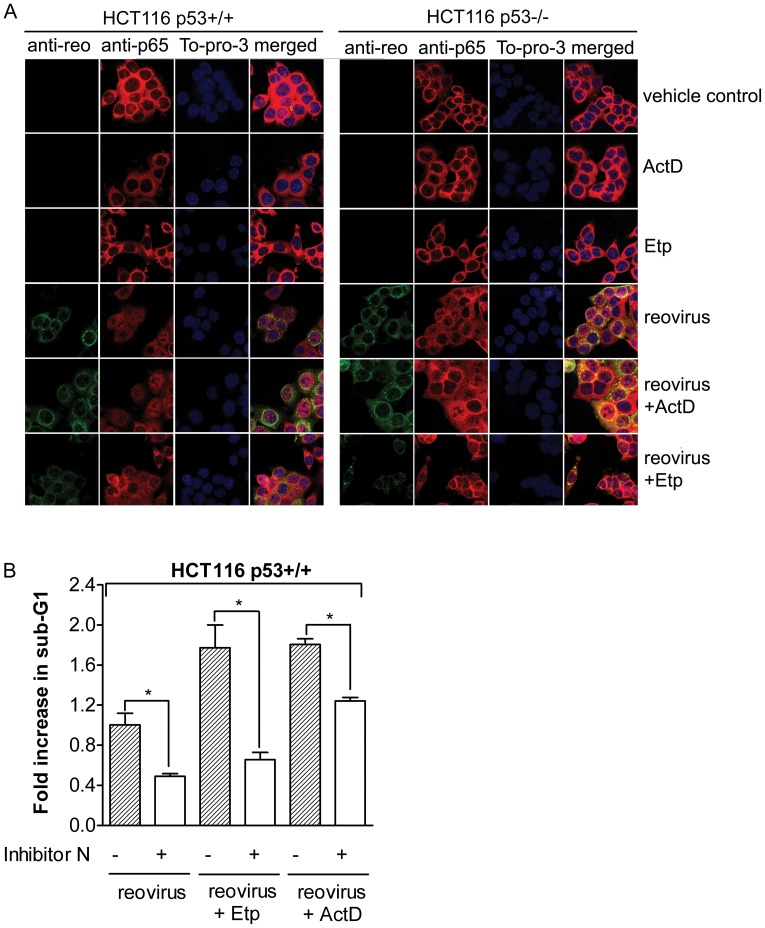
NF-κB activation is important forenhanced apoptosis induced by reovirus and ActD/Etp. **A. *NF-κB activation was determined by NF-κB p65 subunit nuclear translocation.*** HCT116 p53+/+ and p53−/− cells were infected with reovirus at an MOI of 1 in the presence or absence of ActD (0.63 nM) or Etp (1.6 µM) treatment. Cells were fixed at 12-h post ActD/Etp treatment and cells were stained with anti-p65, anti-reo antibody and To-pro-3 for nucleus staining. **B.** ***NF-κB inhibitor N significantly reduces cell death induced by the combination of reovirus and ActD/Etp.*** HC116 p53+/+ cells were treated with NF-κB inhibitor N for 1 h before reovirus infection in the absence or presence of either ActD (0.63 nM) or Etp (1.6 µM) treatment. Cells were collected at 24 hpi for PI staining and the percentage of sub-G1 for each sample was normalized to reovirus infection alone (±*s.e.m.*, n = 3). Student’s *t*-test was used to compare two groups of data; *p<0.05.

p53 accumulation caused by Nutlin-3a treatment enhances reovirus-induced activation of NF-κB [23]. Since the chosen concentrations of ActD or Etp, despite causing minimum cell death, can induce p53 accumulation, it would be interesting to determine whether activation of p53 by a low concentration of ActD/Etp also induces higher levels of NF-κB activation. Nuclear translocation of the p65 subunit of NF-κB was used as an indicator for NF-κB activation. As shown in Fig. 5A, treatment with ActD or Etp of either HCT116 p53+/+ cells or p53−/− cells did not induce noticeable nuclear translocation of p65. Reovirus induced NF-κB activation (nuclear translocation) in both HCT116 p53+/+ and p53−/− cells. However, this activation was further enhanced by the ActD or Etp treatment in HCT116 p53+/+ cells, as p65 distribution was further shifted to the nucleus in HCT116 p53+/+ cells but not in p53−/− cells.

To determine whether the augmentation of NF-κB activation was required for the enhancement of apoptosis induced by the combination of reovirus and ActD/Etp, a NF-κB inhibitor (NF-κB inhibitor N) was used to block NF-κB activation. As shown in Fig. 5B, the level of cell death induced by either reovirus alone or reovirus and ActD/Etp was significantly reduced when NF-κB activation was blocked by the NF-κB inhibitor N. Hence, NF-κB activation is required for the enhancement of cell death induced by the combination of reovirus and ActD or Etp.

## Discussion

In this study, we investigated the possible involvement of p53 when reovirus was combined with traditional chemotherapeutics and whether sub-lethal concentrations of these traditional chemotherapeutics can enhance reovirus-induced cancer cell death. We show here that very low concentrations of ActD or Etp enhance reovirus-induced apoptosis in HCT116 p53+/+ cells but not in p53−/− cells, whereas the same drugs, when used singly, do not trigger significant levels of apoptosis (sub-lethal concentrations). Other chemotherapy drugs such as Dox, at the concentration tested, enhances reovirus-induced cell death in both p53+/+ and p53−/− cells. The reason why Dox is not as effective as ActD/Etp in enhancing reovirus-induced cell death is unclear, but could be due to its complex interactions with the macromolecules in cells, which makes it less dependent on p53 activation. Therefore, information on whether a chemotherapy drug can enhance reovirus-induced apoptosis, coupled with the p53 status of the tumor, would be useful for the selection of suitable drugs and the optimal dosages required for effective combination treatment.

Our results show that ActD and Etp have similar mechanisms for promoting reovirus-induced apoptosis as both involve the enhancement of expression of *p21* and *bax*, and that knocking out either reduces cell death. While the elevation of Bax levels is compatible with enhanced apoptosis (and hence virus spread), the role of p21 in this aspect is less clear as p21 is believed to be anti-apoptotic. A most logical explanation is that *p21* upregulation is a universal DNA damage response when cells harboring wt p53 are exposed to sub-lethal DNA damage conditions. This in turn prolongs the survival of reovirus-infected cells, allowing the virus to fully exploit cellular resources for its own replication. Subsequent virus-induced apoptosis would still be largely dependent on Bax, as a higher Bax level from the combination treatment enhances cell death, and virus cell-to-cell spread.

NF-κB is an important transcription factor that is involved in inflammation and innate immunity. NF-κB also induces cellular alterations that promote tumor formation [33] and is constitutively activated in a wide range of human cancers [34]. Importantly, NF-κB has also been identified as one of the critical mediators for cancer cell chemo- or radiotherapy resistance [35]. For example, the chemoresistance of CEM human T leukemic cells has been attributed to the induction of *p21* by NF-κB [36]. Addition of Etp to these cells induces higher levels of *p21* and leads to increased chemoresistance [37]. Our data shows that reovirus infection alone can induce activation of NF-κB, which is further enhanced by ActD or Etp. NF-κB activation is apparently beneficial to reoviral oncolysis (presumably by upregulating *p21*) as inhibition of NF-κB by the NF-κB inhibitor significantly reduces reovirus-induced cell death. An interesting corollary would be that tumors that are chemoresistant due to inherently high levels of NF-κB would be particular susceptible to reovirus combination therapy.

It is noteworthy that enhancement of reovirus oncolysis by ActD/Etp can only be achieved for target cells harboring wild type p53. Since over 50% of human cancers contain p53 mutations [38], reactivation of mutant p53 or restoration of active conformation of p53 would be required for this combination therapy to work to its full potential. In this regard a number of low molecular weight compounds have recently been developed and shown to be capable of reactivating mutant p53 and restoring wt p53 function [39], [40]. Therefore these small molecules by themselves have anti-tumour activity. Based on observations presented in our present study, it would seem reasonable to suggest that a combination of reovirus, small molecules that restores wt p53 conformation/activity, and sub-lethal dosages of chemotherapeutic drugs such as ActD and Etp would result in the best outcome in terms of comprehensive cancer treatment.

## Supporting Information

Figure S1 **Cell viability assay of DLD1 cells.** **A.** Human colon adenocarcinoma DLD1 cells were infected with retroviruses containing either scrambled sequence or p53 specific shRNAmir sequence (control and p53kd) prepared as described before (23). After selection, cells were treated with reovirus or the combination of reovirus and chemotherapeutic drugs (Dox 0.3 µM, Etp 4.8 µM and ActD 1.89 nM). Cell viability assay was conducted as described in Materials and Methods. Data are means and standard errors of four independent experiments, performed in triplicate. Student’s *t*-test was used to compare two groups of data; *p<0.05, **p<0.001. **B.** Western blot analysis of DLD1 control and p53kd cells when treated with chemotherapeutic drugs (Dox 0.3 µM, Etp 4.8 µM and ActD 1.89 nM). Cells were treated for 24 hours before being harvested.
(TIF)

Figure S2 **Percentage of infected HCT116 cells by reovirus.** HCT116 p53+/+ and p53−/− cells were infected at MOI of 1. Cells were collected at indicated hour-post-infection (control, 6, 12 and 24 hpi) and stained with anti-reovirus antibody. Percentage of infected HCT116 cells was determined by FACS analysis.
(TIF)
