# Supplementary figures and images for: Activation of p53 by Chemotherapeutic Agents Enhances Reovirus Oncolysis
Source: PLoS One. 2013 Jan 16;8(1):e54006. doi: 10.1371/journal.pone.0054006 (PMC3546971; doi:10.1371/journal.pone.0054006)

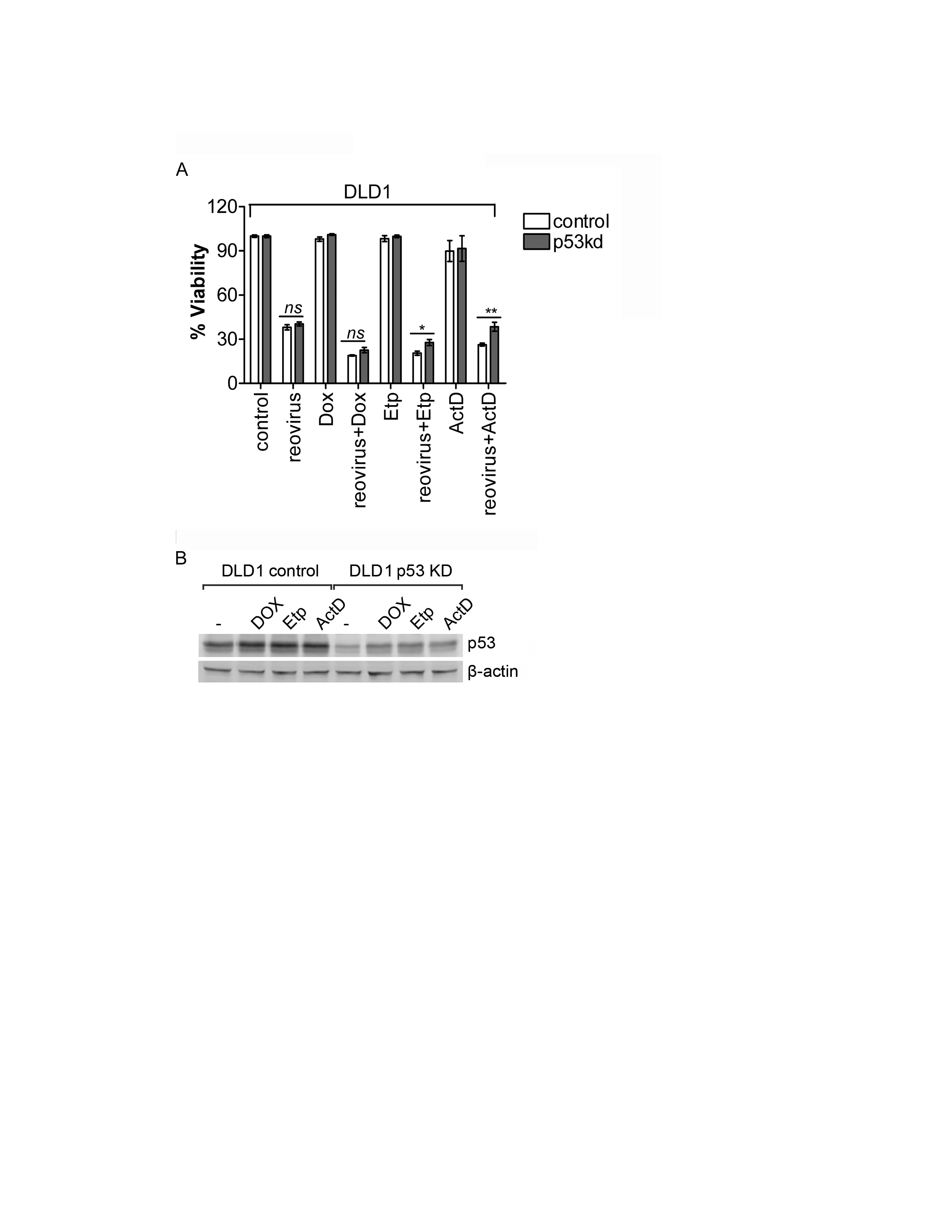

Supplement: Figure S1 — Cell viability assay of DLD1 cells. A. Human colon adenocarcinoma DLD1 cells were infected with retroviruses containing either scrambled sequence or p53 specific shRNAmir sequence (control and p53kd) prepared as described before (23). After selection, cells were treated with reovirus or the combination of reovirus and chemotherapeutic drugs (Dox 0.3 µM, Etp 4.8 µM and ActD 1.89 nM). Cell viability assay was conducted as described in Materials and Methods. Data are means and standard errors of four independent experiments, performed in triplicate. Student’s t-test was used to compare two groups of data; *p<0.05, **p<0.001. B. Western blot analysis of DLD1 control and p53kd cells when treated with chemotherapeutic drugs (Dox 0.3 µM, Etp 4.8 µM and ActD 1.89 nM). Cells were treated for 24 hours before being harvested. (TIF) [file pone.0054006.s001.tif]

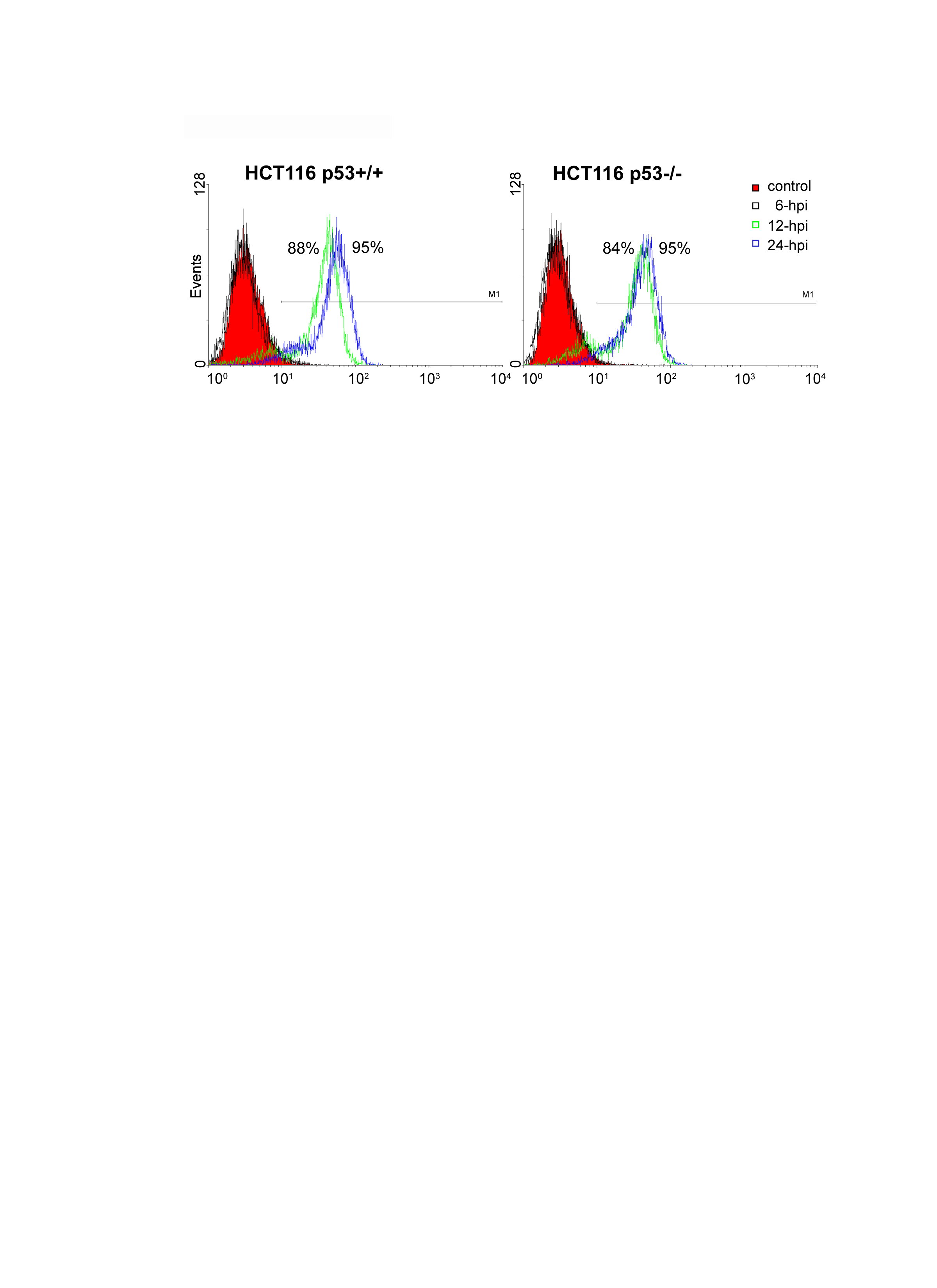

Supplement: Figure S2 — Percentage of infected HCT116 cells by reovirus. HCT116 p53+/+ and p53−/− cells were infected at MOI of 1. Cells were collected at indicated hour-post-infection (control, 6, 12 and 24 hpi) and stained with anti-reovirus antibody. Percentage of infected HCT116 cells was determined by FACS analysis. (TIF) [file pone.0054006.s002.tif]
